# Disclosing crystal nucleation mechanism in lithium disilicate glass through molecular dynamics simulations and free-energy calculations

**DOI:** 10.1038/s41598-020-74764-9

**Published:** 2020-10-20

**Authors:** Federica Lodesani, Maria Cristina Menziani, Kei Maeda, Yoichi Takato, Shingo Urata, Alfonso Pedone

**Affiliations:** 1 Department of Chemical and Geological Sciences, University of Modena and Reggio Emilia, via G. Campi 103, 41125 Modena, Italy; 2 Materials Integration Laboratories, AGC Inc., Yokohama, Kanagawa 221-8755 Japan; 3 Innovative Technology Laboratories, AGC Inc., Yokohama, Kanagawa 221-8755 Japan

**Keywords:** Materials science, Soft materials, Theory and computation

## Abstract

Unraveling detailed mechanism of crystal nucleation from amorphous materials is challenging for both experimental and theoretical approaches. In this study, we have examined two methods to understand the initial stage of crystal precipitation from lithium disilicate glasses using molecular dynamics simulations. One of the methods is a modified exploring method to find structurally similar crystalline clusters in the glass models, enabling us to find three different embryos, such as Li_2_Si_2_O_5_ (LS_2_), Li_2_SiO_3_ (LS) and Li_3_PO_4_ (LP), in the 33Li_2_O·66SiO_2_·1P_2_O_5_ glass (LS_2_P1), in which P_2_O_5_ is added as a nucleating agent. Interestingly, LS_2_ and LP crystals were found inside the LS_2_P1 glass while LS crystal appeared on the glass surface, which agrees with experimental observations. The other method is free energy calculation using a subnano-scale spherical crystal embedded in the glass model. This method, which we called Free-Energy Seeding Method (FESM), allows us to evaluate free energy change as a function of crystal radius and to identify critical size of the crystal precipitation. The free energy profiles for LS and LS_2_ crystal nuclei in the LS_2_ glass models possess maximum energy at a critical radius as expected by classical nucleation theory. Furthermore, the critical radius and the energy barrier height agree well with recent experimental investigation, proving the applicability of this method to design glass–ceramics by atomistic modeling.

## Introduction

Crystal nucleation is a ubiquitous phenomenon and highly relevant to many industrial and technological applications (e.g., bone formation, pharmaceuticals, meteorology, and metallurgy)^1–3^. In glass science, it is the initial stage of glass devitrification (uncontrolled crystallization) and glass–ceramics formation. Devitrification, mostly observed on the glass surface, is an undesired phenomenon in glass manufacturing because the formation of crystals in the glass products often deteriorates their transparency and strength. In the case of nuclear waste vitrification, radiogenic heat induces the formation of soluble crystalline phases^4,5^, resulting in radioactive pollution due to the leakage of radioactive waste from the glassy matrix. On the other hand, technologies for controlling crystallization have contributed to society since they enable us to develop glass–ceramics products with exceptional optical and mechanical properties^6^. Knowledge on the mechanism of the process of crystal nucleation and crystallization in oxide glass is thus fundamental to design a glass–ceramic system that exactly fulfils the requirements for any applications.

Crystal nucleation occurs with appreciable rate when the glass is heated and held near the glass-transition temperature (*T*_g_). The kinetics and thermodynamics of crystal nucleation are usually explained by the Classical Nucleation Theory^7–10^ (CNT), which simplifies the initial germ of nucleation as a spherical shape of radius *r* since it may minimize the surface energy between a crystal and the supercooled liquid. The other assumption of CNT is that an initial germ of nucleation has the same macroscopic properties, such as density, structure, composition and thermodynamic properties, with those of a stable crystalline phase to be formed.

In this framework, the nucleation rate^10^ is given by Eq. 1:

1 $$ I = I_{O} exp\left( { - \frac{{W^{*} + \Delta G_{D} }}{{k_{B} T}}} \right), $$

where $I_{O}$ is the pre-exponential term, $k_{B}$ and *T* are the Boltzmann constant and temperature. $\Delta G_{D}$ is the kinetic contribution to the nucleation rate, that is, the activation energy for the transfer of species through the melt/nucleus interface, and $W^{*}$ is the free energy change due to the formation of a new nucleus of critical radius r*. Assuming that, the atomic clusters or embryos below the critical size are not stable and can be dissolved into the original melt without producing a nucleus. Once an embryo overcomes the critical size, it can grow to form a crystalline phase.

Even though the CNT is widely accepted as a fundamental theory, unraveling the origin of the crystal nucleation mechanism in glass forming liquids is still a challenging issue in glass science community. The nucleation mechanism is categorized into two main types: one is homogeneous nucleation and the other one is heterogeneous one^11^. The heterogeneous nucleation at surfaces and interface boundary between different materials is the most common mechanism since it requires lower activation energies to occur. Indeed, a variety of nucleating agents (i.e. metal oxides such as ZrO_2_, TiO_2_ and CeO_2_^12^, non-metal oxides^13,14^ like a P_2_O_5_ and colloidal metal nanoparticles^15^) are used to accelerate bulk crystallization.

Contrarily, the homogeneous nucleation rarely occurs because of the higher activation energy since the nucleus forms from the bulk without the help of any phase inhomogeneities or boundaries. However, in the case of homogeneous nucleation, the nuclei form throughout the entire glass matrix, allowing a better control of the processing conditions and of the final glass–ceramic microstructure and properties. The homogeneous crystallization is usually associated with the similarity of microstructures between glass and crystals. Nevertheless, the mechanism is not well explained only by the simple assumption^16–18^ because intermediate metastable phases might also play an important role. It is thus worth investigating the origin of the homogeneous crystallization in glass forming liquids^19^, using theoretical simulations^20,21^.

**Figure 1:**
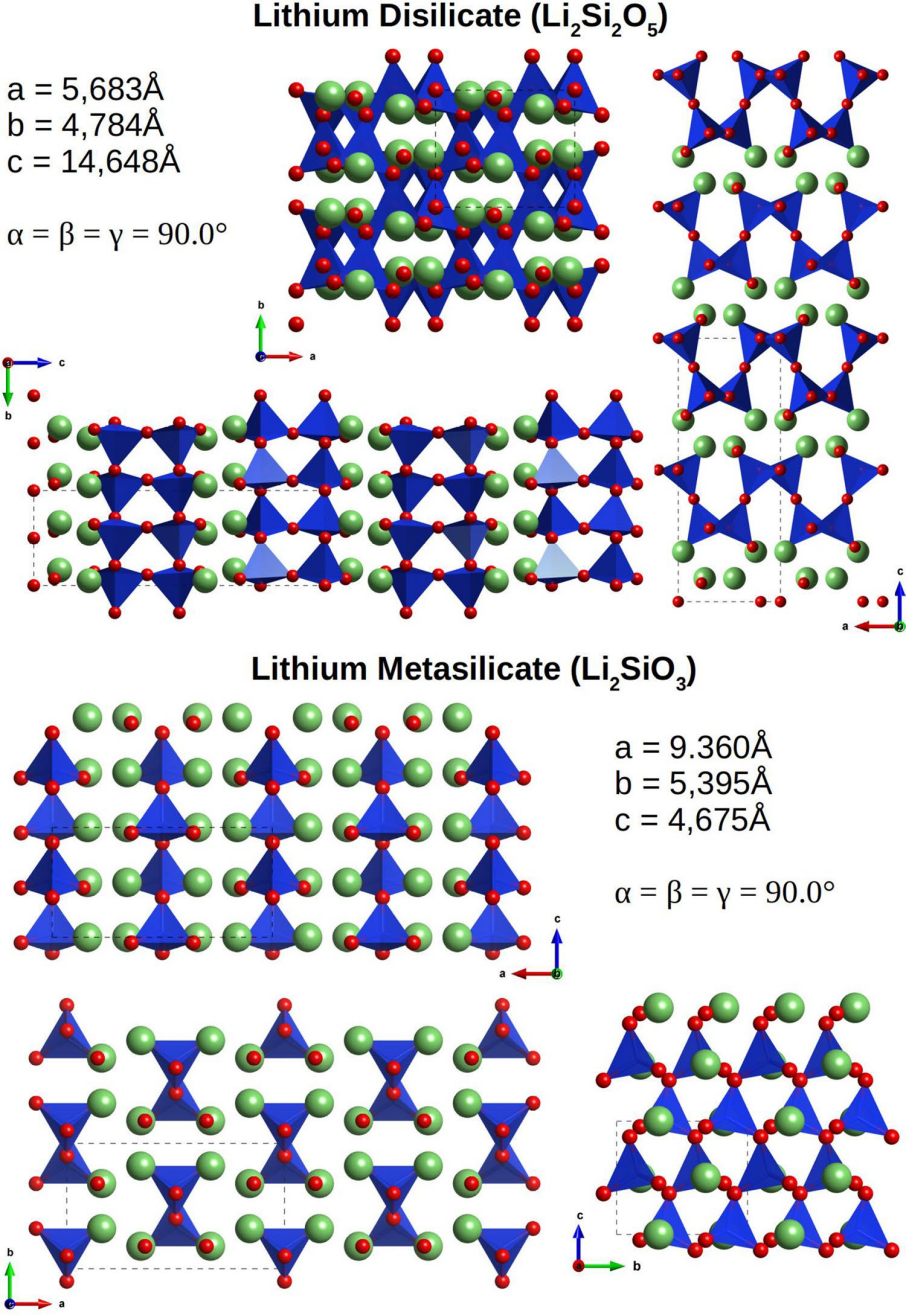
Structure of the LS and LS_2_ crystals along different orientations and unit cell parameters. Blue tetrahedral represents silicon, red and green spheres represent oxygen and lithium ions, respectively.

A typical homogeneous crystallization is observed in the lithium silicate glass system close to the Li_2_O·2SiO_2_ (lithium disilicate, LS_2_) composition^7^, and many experimental investigations using a large variety of techniques^22–29^ have been investigated. Accordingly, two possible phase evolution mechanisms have been proposed^7,28,29^: (1) both LS_2_ and metastable Li_2_O·SiO_2_ (lithium metasilicate, LS) phases simultaneously nucleate homogeneously but the latter phase disappears during the heat treatment or (2) the LS crystal is firstly nucleated and subsequently promotes the heterogeneous LS_2_ crystal on it. The latter hypothesis was suggested by the fact that the CNT underestimates the steady-state nucleation rate in several orders of magnitude. To fill the gap between CNT and experimental observation, it is assumed that the metastable crystalline phase in the early stage would decrease the activation energy. In addition, some experimental groups have observed the formation of metastable phases with stoichiometry close to the LS_2_ composition (called α’-LS_2_ and β’-LS_2_ in ref_._^23^). Meanwhile, others have obtained the LS crystalline phase^27–29^ although the conversion of the LS crystal (if formed before) to the LS_2_ crystal would require a high energetic cost because the two phases have quite different stoichiometry and structures, as shown in Fig. 1 (LS_2_ has a double-layered silica structure with Li ions in the interlayers, whereas LS has a chain-like silica structure surrounded by Li ions).

In spite of the many efforts made in the past, there is still no consensus neither on the precipitation of metastable phases prior to the LS_2_ one, nor on their composition, and thus the detailed process of the homogeneous crystallization is still open to question. In this work, we employ Molecular Dynamics (MD) simulations coupled with an original algorithm to search crystal-like structures in amorphous models and Free-Energy calculations on a crystal-embedded in a glass model in order to answer the following queries: (*i)* Is homogeneous nucleation in oxide glasses associated with the existence of pre-formed nuclei (embryos) whose stoichiometry and structure are similar to those of the crystals precipitating? (*ii)* Does the LS crystal form before the LS_2_ one inside the glass or on the glass surface? (*iii)* How do temperature and P_2_O_5_ nucleating agent affect the nucleation? (iv) Does the nucleation of the LS_2_ and LS phases obey the Classical Nucleation Theory?

It is worth noting that although, as stated before, nucleation and crystallization occur (with appreciable velocity) from the melt rather than the glass (defined as the undercooled liquid under *T*_g_) the latter has the same structure of the undercooled melt (this is especially true for MD-derived glass models). Therefore, in this work we have sought the fingerprints of nucleation analyzing computational glass bulk and surface models with compositions 33.3Li_2_O·66.7SiO_2_ (LS_2_ Glass) and 33Li_2_O·66SiO_2_·1P_2_O_5_ (LS_2_P1 Glass) instead of melts. Moreover, it is also worth to note that the high viscosity of the oxide glasses investigated here does not allow to follow the dynamic evolution of nucleation and crystal growth with unbiased MD simulations within a reasonable simulation time, contrary to model systems^30,31^.

### Computational details

The LS_2_ and LS_2_P1 glasses were generated using MD simulations by employing the modified PMMCS force-field published in ref.^32^ and described in the ESI. The LS_2_P1 composition was investigated to understand the effect of P_2_O_5_ on the structure and nucleation of Li_2_Si_2_O_5_ and Li_2_SiO_3_ crystals in the glass since it is well known that addition of P_2_O_5_ favors the precipitation of the LS_2_ crystal and the formation of glass–ceramics^13,33^.

Glass structural models containing 13,500 and 12,160 atoms were generated for LS_2_ and LS_2_P1 glasses, respectively, through the melt and quench approach by MD simulations^34^. Four replicas of each glass model have been examined to confirm the reproducibility of the results and to estimate the variability in the glass properties. The leap-frog algorithm encoded in the DL_POLY2.14 package^35^ was used to integrate the equation of motions with a time step of 2 fs. The initial configurations were generated by randomly placing the atoms in a cubic box, whose size corresponds to the experimental density. Table S1 lists the number of the atomic species and the experimental density used to determine the unit cell size (5.4 nm for LS_2_ and 5.2 nm for LS_2_P1).

The systems were heated and held at 3500 K for 100 ps, which is sufficient to melt the samples and remove the memory of the initial configurations. The liquids were then monotonically cooled to 300 K with a cooling rate of approximately 5 K/ps. The cooled glass structures were subjected to a final equilibration run of 200 ps. In these cases, the canonical ensemble (NVT) was employed, and Berendsen thermostat^36^ was used to control the temperature (frictional constants set to 0.2 ps). The coulomb interactions were calculated by the Ewald summation method with a cutoff distance of 12 Å. The short-range interactions were evaluated using cutoff values of 5.5 Å.

In addition to the bulk glass models with periodic conditions, slab models with two surfaces were created by eliminating periodic boundary conditions in the z-axis. The slab models were replicated in x-axis by one time in order to have larger surface areas and thus more statistics, then the systems were heated from 300 to 1800 K with a heating rate of 5 K/ps and cooled down at 300 K with the same cooling rate to relax the surface structure. It is worth to note that the melting temperature of the LS_2_ crystal computed by heating the system in the isobaric-isothermal ensemble (NPT) with the same rate (5 K/ps) is around 1750–1800 K, whereas the glass transition temperature is around 1250–1300 K. Both values overestimate the experimental data (T_m_: 1306 K, T_g_: 727 K^17^) because of the application of periodic boundary conditions, which eliminate surfaces where melting is initiated in real samples, and the huge cooling rate used in computer simulations with respect to the one used in experiments, as discussed in ref.^37,38^.

#### Cluster analysis: the algorithm

The aim of this analysis is to explore atomic aggregates whose stoichiometry and structure are similar to a particular crystalline phase that is thought to nucleate and crystallize from the glass forming liquid. The cluster analysis has been performed using a new FORTRAN90 code based on the one developed in the past by Pedone^18^ (Cluster code). The program analyzes the glass structure generated through MD simulations and systematically samples the stoichiometry and local structure around each atomic species (for example lithium, oxygen or silicon in lithium silicate glasses). Then, it compares the extracted clusters with a reference crystal phase (Li_2_Si_2_O_5_ for example), which is expected to form in the glass.

A similar approach, known as the Adaptive Template Method, has been applied to identify crystal lattice types (FCC, BCC and HPC) in hard sphere systems^39^. In our algorithm, a similar idea with the Adaptive Template Method was combined with our original Cluster code^18^, which enables us to investigate more complicated crystals with multiple species in multicomponent glasses. Here, we introduce the detailed procedure of the extended algorithm.

The program performs the following steps:

1. Read a trajectory file of MD simulations (in this work, DL_POLY REVCON file) and a crystal structure with a unit cell (P1 symmetry) to be explored. Then, specify a reference atom of the crystal structure.
2. Count the number of each atomic species within a spherical region with radius *r*_k_ around the reference atom of the crystal structure. The numbers of species are stored in a reference vector **R**_A_, which is noted as (N_Li_, N_Si_, N_O_) for the LS_2_ glass, for example. In the case of the LS_2_P1 glass, the reference vector **R**_A_ has one more dimension as (N_Li_, N_Si_, N_O_, N_P_).
3. Subsequently, put a center of a spherical probe on an atom whose atomic type is the same with the reference atom in the MD trajectory. Then, count the number of each species in the spherical probe with a radius *r*_k_, which defines a vector ***χ***_A,j_ for atom *j*.
4. Evaluate the Hamming distance $\delta_{j} \left( {r_{K} } \right) = \left| {R_{A} \left( i \right) - \chi_{A,j} \left( i \right)} \right|$ to measure the similarity of the local structure in the MD trajectory to the reference crystal.
5. Repeat the steps from #2 to #4 with varying the proof radius *r*_k_ from a minimum (*r*_min_) to a maximum (*r*_max_) value every small increment (*dr*). Then, evaluate the cumulative distance for atom *j* at the radius *r*_k_ is computed, as $\Delta_{j} \left( {r_{k} } \right) = \sum\nolimits_{{r_{min} }}^{{r_{k} }} {\delta_{j} } \left( {r_{k} } \right)$. The atom with smaller cumulative displacement is judged to possess more similar microstructure to that of the crystal explored.

At the end the code outputs: i) PDB files with atomic structure of the best cluster found for each radius; ii) a statistic on the best clusters found at each radius and a distribution of the cumulative distances of all the target species in the glass.

It is worth to note that when the increment *dr* is small (i.e. 0.1 Å), the cumulative distance of atom *j* $\Delta_{j} \left( {r_{k} } \right)$ gives an idea of the degree of matching between the total distribution function of the reference atom in the crystal and the total distribution function of each atom in the glass. In this work, the analysis has been carried out using *r*_min_ = 2.0 Å, *r*_max_ = 6.0 Å, dr = 0.2 Å without considering oxygen atoms.

#### Free-energy seeding method (FESM)

In addition to the morphological analyses, we have also evaluated the thermodynamics on the nucleation of both the LS and LS_2_ crystals inside the LS_2_ glass. To determine the work necessary to create a critical nucleus of such crystals in the LS_2_ glass, the Free-Energy Seeding Method (FESM) has been used. This method inserts a cluster of a given shape into the supercooled liquid, as the seeding method proposed by Espinosa et al.^30^ but determines the thermodynamic and kinetic parameters that characterize the nucleation process differently. In the original seeding method, the critical nucleus is determined by following the dissolution or growth of the inserted clusters in the fluid; the thermodynamic and kinetic parameters, such as the interfacial free energy, the chemical potential difference between crystal and liquid phases, and the attachments rates of particles to the critical cluster, are determined independently. This interesting method is well-examined and a reasonable choice to low viscous systems or model systems, such as Lennard–Jones, Hard Sphere and spherical coarse-grained water models, whereas it is impractical for high viscous and complex systems like multicomponent silica-based glass forming liquids. Indeed, we conducted several test simulations on the LS_2_ system but could not observe crystal growth for any nucleus sizes in the timescale accessible to classical MD simulations (up to 1 µs), even though it is possible to observe dissolution of the crystal nuclei at temperature above the computational glass transition temperature (> 1300 K in this case).

Therefore, to apply the idea analogous to the seeding method to viscous glass melt, here we simplified the approach. In the case of FESM, we do not simulate dissolution and nucleation, explicitly, even though our approach also embeds a spherical nucleus of the LS or LS_2_ crystal with different radii into a fully explicit lithium disilicate (LS_2_) glass melt. Instead, we evaluated the energy profile as a function of the cluster radius. From this energy profile, it is possible to extract the nucleation activation energy, the critical size of the cluster, and the interfacial free energy, which can avoid the possible timescale problem to observe dissolution and growth in our highly viscous systems.

The embedded systems have been built as follows. First, we built a crystal model. For the modeling of LS_2_ nucleus in the LS_2_ glass, we replicated the unit cell of the LS_2_ crystal 10 × 10 × 4 times to generate a box containing 14,400 atoms, and the side lengths of the simulation box varied accordingly to the experimental density of the LS_2_ glass. On the one hand, to model the LS nucleus in the LS_2_ glass matrix, after replicating the lithium metasilicate unit cell by 5 × 10 × 8 times to create a supercell with 9600 atoms, subsequently, the simulation box was enlarged to have the same density of the LS_2_ glass. Then, 1600 SiO_2_ formula units were randomly added in the vacuum spaces in the simulation box to maintain the overall compositions and density same as those of LS_2_ glass. It is worth to mention that system size effect is presumable in these calculations, and thus, we restricted the volume of the embedded crystal nucleus less than 10% of the total simulation box to avoid the artifact.

Finally, in both cases, an atom was randomly chosen and the positions of the atoms locating within a distance *r*_sphere_ from the central atom were fixed as an embedded crystal cluster (seed). Then, the cluster embedded systems were melted and quenched using the same procedure employed to generate the bulk structural models with fixing the embedded crystal, generating a model with a crystal embedded in a glassy matrix. Finally, the model was equilibrated for 2.4 ns at 800 K and 1000 K to investigate the temperature effect. During this equilibration, the positions of the atoms of the embedded crystals were also relaxed without constraints. Nuclei with r_sphere_ from 6 to 14 Å were created and 7 replicas for each system were examined to have a sufficient statistic. An example of the starting and final structures for both the LS_2_ and LS crystal nuclei into the LS_2_ glass is shown in figure S1 of the ESI.

To evaluate the energy differences between crystal, glass, and the cluster-embedded models, MD simulations on the LS_2_ glass and LS_2_ crystal were also performed (four replicas for each). For all the aforementioned systems, the internal energy ($E_{S}$) of the system was accumulated and the Helmholtz free energy ($A_{S}$) of the state S (S refer to the crystal, glass and embedded system states) computed using the thermodynamic formulas^36^

2 $$ A_{S} = - k_{B} Tln\left\langle {{\text{e}}^{{ - \frac{{E_{S} }}{{k_{B} T}}}} } \right\rangle $$

The free energy of nucleation (W) with cluster radius *r* is then computed as

3 $$ W\left( r \right) = A_{CryGlass} \left( r \right) - A_{Glass} $$

where *A*_CryGlass_ and *A*_Glass_ are Helmholtz free energy of the crystal embedded system and the glass, respectively. In our calculations, we used the Helmholtz free energy of the more stable glass as a reference. In CNT, the free energy of nucleation of a spherical nucleus with negligible strain energy is assumed to be composed of surface free energy and volume free energy as follows:

4 $$ W = 4\pi r^{2} \gamma_{sl} - \frac{4}{3}\pi r^{3} \Delta A_{V} , $$

where $\Delta A_{V}$ is the free energy difference between the melt and the crystal per unit volume of the crystal, whereas $\gamma_{sl}$ is the interfacial free energy per unit area required to create a liquid/crystal nucleus interface. The interfacial free energy, which governs largely the crystal nucleation rate, is assumed to be equal to that of a planar interface and thus to be independent on the nucleus size. This is known as the capillary approximation and is one of the most serious shortcomings of CNT. A benefit of our approach is that the interfacial free energy is not an input parameter independent of the nucleus size and temperature but is a property that varies inherently with the nucleus dimension and the temperature. The interfacial free energy can be computed from the MD simulations as:

5 $$ \gamma_{sl} \left( r \right) = \frac{{W\left( r \right) + \frac{4}{3}\pi r^{3} \Delta A_{V} }}{{4\pi r^{2} }}. $$

where $\Delta A_{V} = A_{Crystal} - A_{Glass}$ at a defined temperature. We used T = 800 K in this work to make our results comparable to that reported by McKenzie et al.^40^ (that used a hybrid MD/MC/implicit approach to compute the free energy of nucleation of the LS and LS_2_ crystals inside the LS_2_ glass) and to the experimental data^41^.

It should be noted that since the temperature, 800 and 1000 K, are below the computational glass transition and melting temperatures, the effect of the temperature on the free energy profiles is investigated with assuming that the topology of the crystal/glass interface is similar. In other words, we do not investigate the dynamic evolution of the crystal growth with temperature but generate equilibrated model systems containing a crystal nuclei in an amorphous LS_2_ matrix, as previous Monte Carlo simulations^40^.

## Results and Discussions

### Bulk and surface structures

Homogeneous nucleation is usually associated with the similarity of the short and intermediate-range structures between the glass forming liquid and the crystal that crystallizes from it^16,17,42,43^. Since the short-range structures measured by the cation-oxygen distances, coordination numbers and the O-Si–O, O-Li–O bond angle distributions are not distinguishable between LS_2_ and LS crystals as well as between the crystals and the frozen melts, we only give our attention to the structural differences in intermediate-range order. The intermediate-range structures are described by Li–Li distributions and the Q^n^ distributions, which are the populations of the quaternary species (Q) of the network former cations connected to *n* bridging oxygens (BO), representing degree of polymerization of the network.

**Figure 2:**
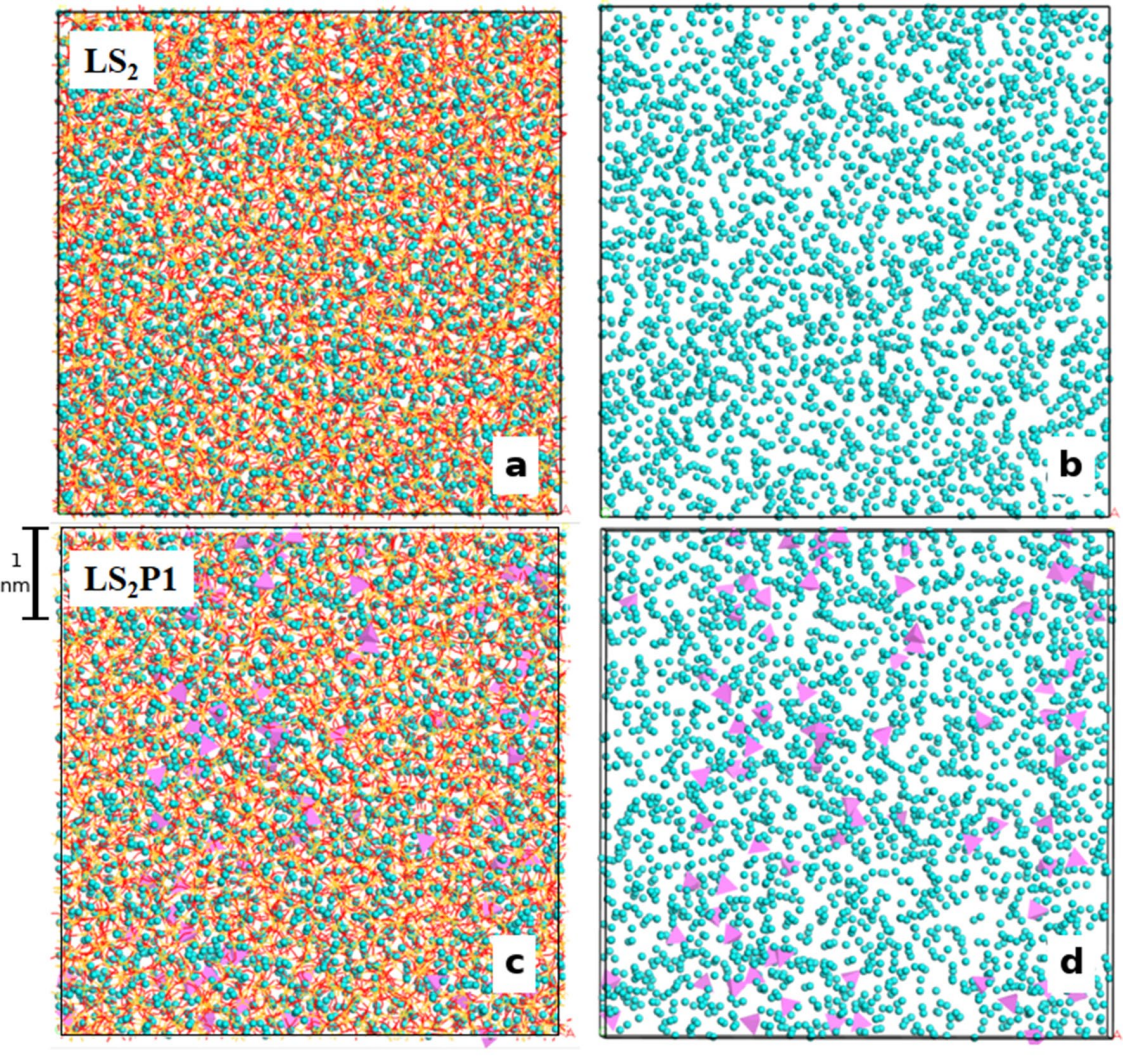
Snapshot of the MD-derived bulk structural model of the LS_2_ glass showing all the ions in the box (**a**) and Li ions only (**b**). Snapshot of the LS_2_P1 glass showing all the ions in the box (**c**) and Li (cyan spheres) and PO_4_ tetrahedra only (**d**).

**Figure 3:**
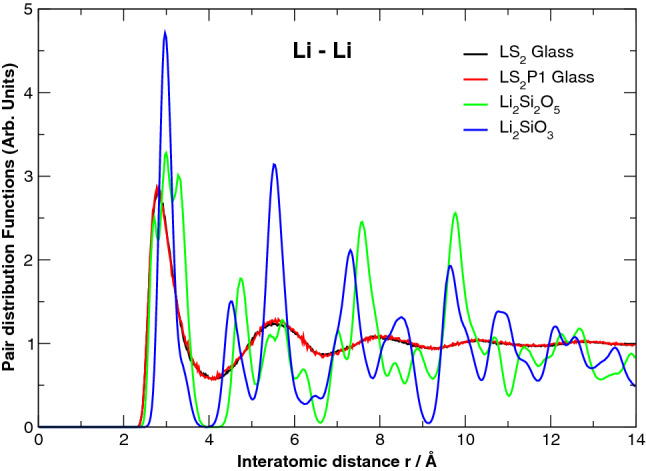
Li–Li Pair Distribution Functions of the LS_2_ and LS_2_P1 Glasses compared to that of the LS_2_ and LS crystals.

Figure 2 shows the bulk structures of the LS_2_ and LS_2_P1 glasses at 300 K. A visual inspection of the atomistic configurations reveals that phosphorous atoms form orthophosphate units and tend to segregate each other. The units attract Li ions, forming Li-richer regions around PO_4_ units. Indeed, the Q^n^ distributions of the P species (not reported) reveals that 98.8% of PO_4_ units are Q^0^ species, in excellent agreement with NMR experiments on similar glasses^27^. Table S4 compares the Q^n^ distribution of silicon and the network connectivity (NC) representing the average number of BO per silicon for the two glasses. More Q^4^ silicon and larger NC of the LS_2_P1 glass reveal that the silica network is slightly more polymerized than the LS_2_ glass. This is a consequence of the existence of the orthophosphate units, which attract Li ions to balance their negative charge, depleting Li ions around the silica network and thus Q^4^ silicon increases. In both glasses, silicon is predominantly present as Q^3^ species as in the case of LS_2_ crystal, which is formed by two tetrahedral layers of silicon (100% Q^3^ species). The amount of the Q^2^ silicon species, which are the majority in the LS crystal, are below 20% in both glasses. The Li-rich regions can be seen in Fig. 2 as a formation of percolation channels of Li ions; these are highlighted by the worm-like Li distribution sandwiched between chains and sheets of silica. An extended ordering over four alkali coordination shells is also revealed by the Li–Li pair distribution function (PDF) drawn in Fig. 3. In this figure, the Li–Li PDFs of LS and LS_2_ crystals at 300 K are also compared, and we can find that the peak positions of both crystals are close to the four peaks in the PDF of the glasses. However, the intensities of the first two peaks of the glasses are more similar to those of the LS_2_ crystals.

The other useful parameter for probing structural resemblance or dissimilarity between crystalline and glassy phases is the Li–Li homonuclear *M*_2_ dipolar moment^16^. The *M*_2_ dipolar moment is related to the Li–Li distance correlation and can be an ability index of a glass to be transferred to a crystal with the same stoichiometry. It is computed using the van Vleck equation:^44^.

6 $$ M_{2, Li - Li} = 0.9562\frac{1}{{N_{Li} }}\left( {\frac{{\mu_{0} }}{4\pi }} \right)^{2} \gamma_{Li}^{4} \hbar^{2} \mathop \sum \limits_{i = 1}^{{N_{Li} - 1}} \mathop \sum \limits_{j = i + 1}^{{N_{Li} }} r_{ij}^{ - 6} , $$

where $$ N_{Li}$$ is the number of the Li atoms in the simulation box, $$\mu_{0}$$ is the vacuum permittivity, $$\gamma_{Li}$$ is the gyromagnetic ratio and $$r_{ij}$$ is the distance between atoms *i* and *j*. The calculated $$M_{2,LiLi}$$ for the LS_2_ and LS_2_P1 glasses are of 49.7 and 49.6 × 10^6^ rad^2^ s^−2^ and closer to that of the LS_2_ crystal ($$M_{2,LiLi} = 57.8$$ × 10^6^ rad^2^ s^−2^) than the LS crystal ($$M_{2,LiLi} = 75.7$$ × 10^6^ rad^2^ s^−2^). Accordingly, it seems unlikely that the LS crystal can nucleate homogeneously from the bulk of the melt, as suggested in some of the previous experimental^28^ and computational works^40^.

### Nucleus of crystals in bulk and on surface

Another hypothesis to explain and predict the crystallization of a specific phase from a glass forming liquid involves the clustering of specific atoms forming embryos of the crystal with subcritical dimensions. These embryos, formed during the fast quenching of the melt, can trigger the nucleation during heat treatment and be considered as structural markers for the prediction of the crystallization of a particular phase. MD simulation is an exceptional method to detect such extremely small embryos, which is indeed difficult to be found experimentally. To test this hypothesis we performed a cluster analysis^18^ on the bulk structure of the two simulated glasses using the modified Cluster code described before.

**Figure 4:**
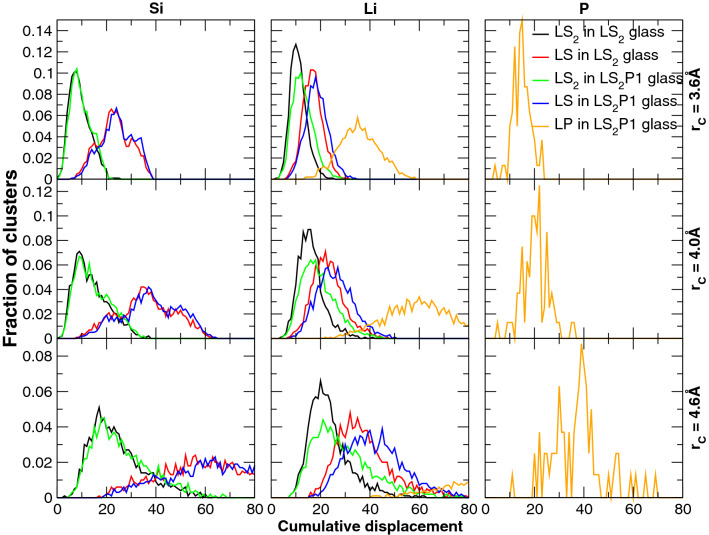
Distribution of cumulative distances (with respect to Li_2_SiO_3_, Li_2_Si_2_O_5_ and Li_3_PO_4_ crystals) of clusters of radii 3.6, 4.0 and 4.6 Å centered on Silicon (left) and Lithium (middle) and Phosphorous (right) for the LS_2_ and LS_2_P1 glasses.

**Figure 5:**
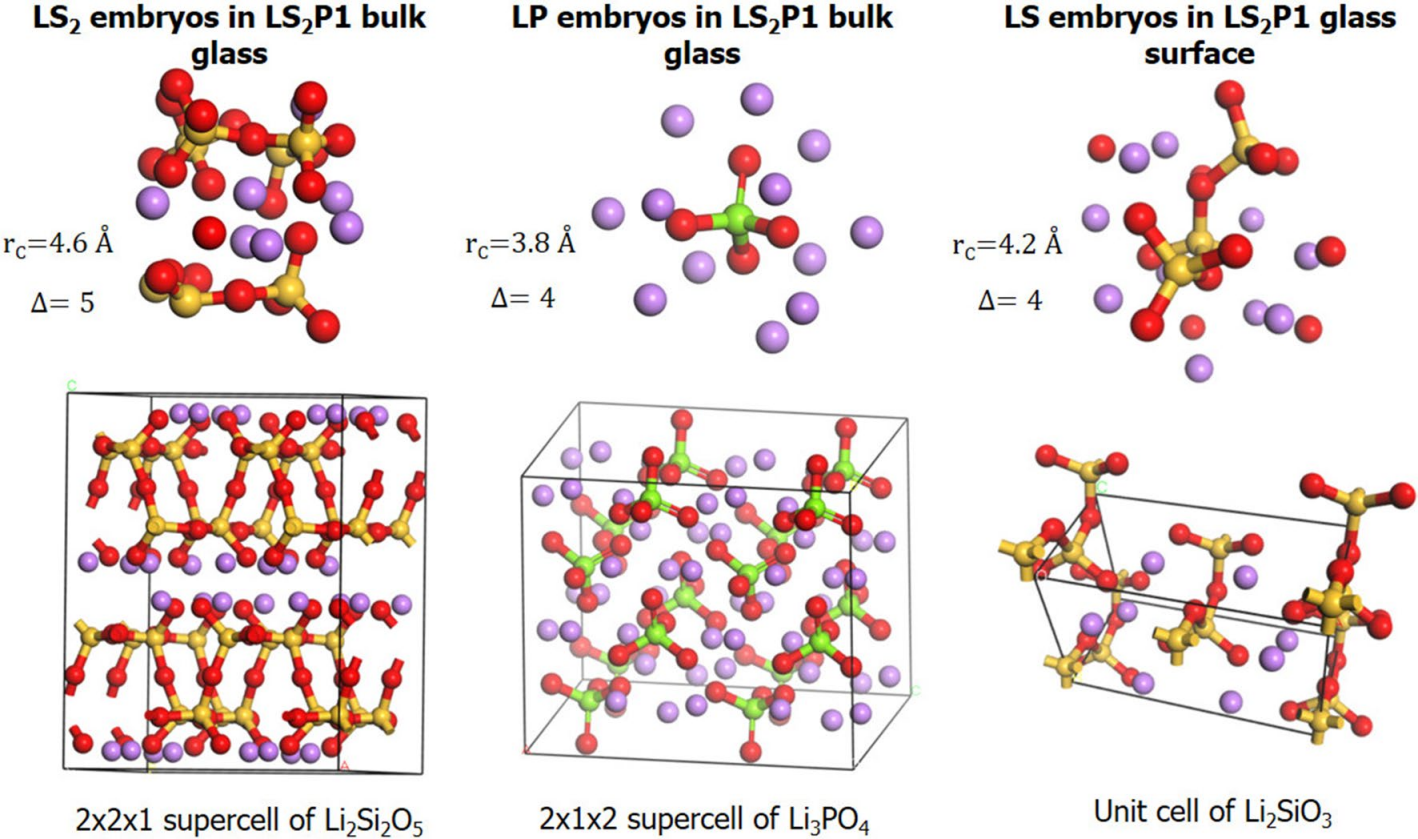
Embryos of Li_2_Si_2_O_5_ (LS_2_), Li_2_SiO_3_ (LS) and Li_3_PO_4_ (LP) found in the LS_2_P1 glass and structures of the three crystals.

Figure 4 reports the distribution of the cumulative displacements of atomic clusters similar to the Li_2_SiO_3_, Li_2_Si_2_O_5_ and Li_3_PO_4_ crystals in the LS_2_P1 glass within a sphere of radius of 3.6, 4.0 and 4.6 Å centered on Li, Si and P, respectively. The figure shows that silicon and lithium environments in the glass structure are similar to the Li_2_Si_2_O_5_ crystal rather than the Li_2_SiO_3_ one at any radii. However, the discrepancy is more evident for silicon since the two distributions are more separated than the ones for the clusters centered on lithium. It is interesting to note that the distribution of the clusters centered on Li shifts to higher cumulative distances by P_2_O_5_ addition_,_ revealing that the formation of embryos similar to the metasilicate phase is disfavored in the LS_2_P1 glass. Figure 5 shows the most Li_2_Si_2_O_5_ –like embryo obtained by means of a sphere of 4.6 Å radius centered on Li in the LS_2_P1 glass. The embryo contains 7 Li and 6 Si atoms as in the reference cluster of the crystal and shows a layered structure similar to that of the Li_2_Si_2_O_5_ crystal. Moreover, an embryo whose stoichiometry and structure is similar to the Li_3_PO_4_ crystals (see Fig. 5), which is also found in the LS_2_P1 glass when the radius of sphere is 4.0 Å, agrees with the experimental fact that the lithium orthophosphate crystal phase appears as a secondary phase^27^.

**Figure 6:**
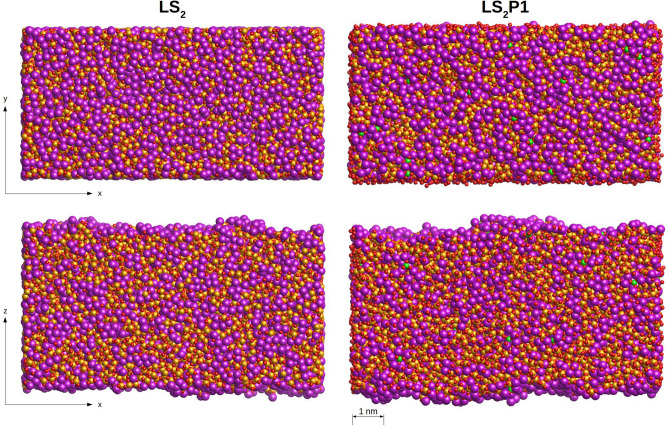
Top and lateral view of the surface of the LS_2_ and LS_2_P1 glasses. Li, Si and O ions are, respectively, represented by violet, yellow and red spheres. Orthophosphate units are represented as green tetrahedral.

**Figure 7:**
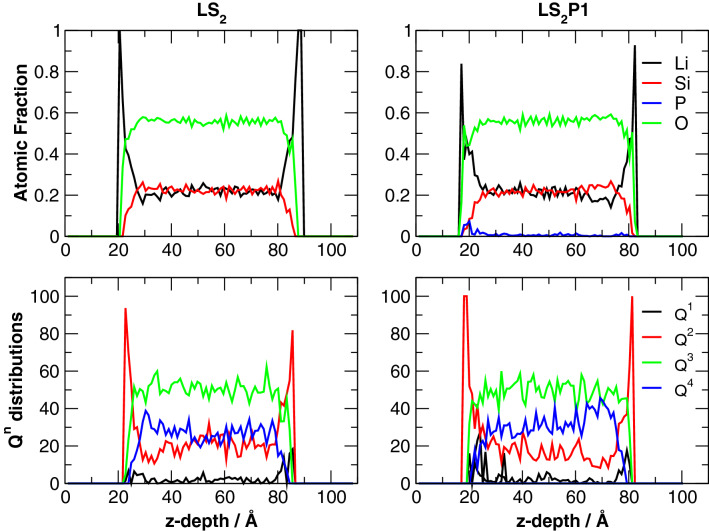
*Z*-depth profiles of the atomic fraction of Li, Si, P and O ions and Q^n^ distributions of Si ions in the LS_2_ and LS_2_P1 glass surface models.

Interestingly, the situation drastically changes at the glass surfaces. Figure 6 shows the top and lateral views of the slab models for the LS_2_ and LS_2_P1 glasses. The figure demonstrates that the surface is much richer in Li ions than the bulk. During the formation of the glass surface the structure undergoes a drastic rearrangement with Li ions (violet spheres) aggregating in layers and forming percolation channels that flow to a surface to the other side. The abundance of Li ions at the glass surface is also confirmed by analyzing the fraction of each element along the *z*-direction as shown in Fig. 7. Indeed, the first atomic layer at the surface is exclusively composed by lithium ions, the second by oxygen ions and the third by silicon ions. The concentration profiles also show that the P ions, albeit present in small quantities, tend to gather at the subsurface rather than in the bulk.

In addition, the Q^n^ distributions of silicon change considerably during the surface formation since the migration of Li ions to the surface leads to breaking of Si–O-Si bonds and reducing the connectivity of silicon. In fact, Fig. 7 reveals that silicon ions at the glass surface are almost exclusively Q^2^ species (~ 80–90% in the LS_2_ and 100% in the LS_2_P1 glass) as in the case of the LS crystal. This finding is extremely interesting and demonstrates that the surface provides favorable stoichiometric conditions for the nucleation and crystallization of the LS crystal rather than LS_2_ one. Indeed, in Fig. 5, an atomic cluster possessing structure and stoichiometry analogous to the LS crystal is found on the LS_2_ glass surface. The cluster is constituted by a chain of three SiO_4_ units surrounded by 10 Li ions, similarly to the chain present in the LS crystal. On the contrary, LS-like embryos with the same cumulative displacements were not found in the bulk of the glass model. It seems thus unlikely that the LS crystal phase nucleates homogeneously from the bulk of the glass in these glasses.

### Free energy of crystal nucleation

**Figure 8:**
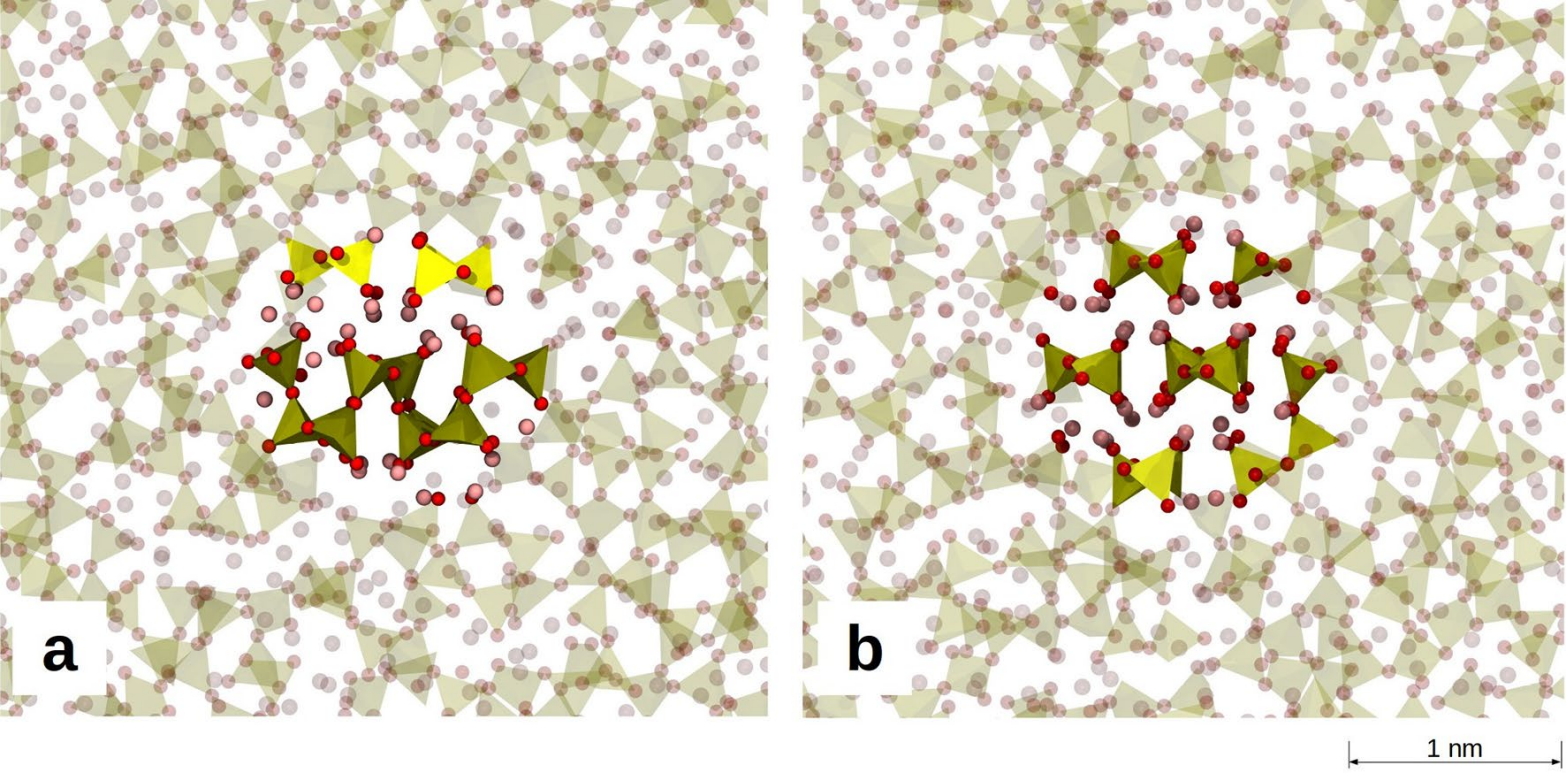
Structure of boxes with critical size nuclei. On the left, glass with LS_2_ composition with the critical nucleus of Li_2_Si_2_O_5_ crystal. On the right, glass with LS_2_ composition with the critical nucleus of Li_2_SiO_3_ crystal.

**Figure 9:**
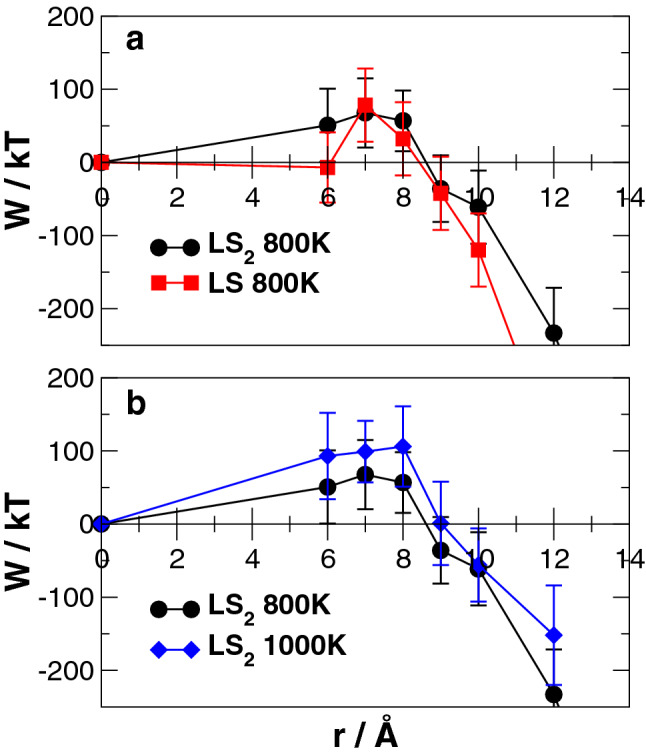
Free energy of nucleation for (**a**) the LS and LS_2_ crystals in the LS_2_ glass at 800 K and (**b**) the LS_2_ crystals in the LS_2_ glass at 800 K and 1000 K with relative standard deviation.

To obtain further insights on the possible mechanisms of nucleation from the LS_2_ glass forming liquid, we computed the Helmholtz free energies of the LS and LS_2_ crystals formation using the FESM method (see Method section). In this free energy calculation, we modeled a spherical crystal nucleus embedded into a glass model as seen in Fig. 8 and examined size dependency of the nucleation free energy for both LS_2_ and LS crystals at 800 K. The Helmholtz free energy profiles are reported in Fig. 9a. The error bar in this figure represents standard deviation of free energies evaluated by seven independent replicas. As expected from CNT, the free energy first increases and then decreases after reaching the maximum energy (barrier for nucleation) at the critical radius. It is worth highlighting that LS_2_ clusters with radius less than 5 Å were not considered to build the free energy profile in Fig. 8 since the free energy of such embryos formed during the glass formation is expected to be almost zero. Indeed, LS clusters of such small sizes lost the crystalline structure during relaxation, which indicates the instability. According to the figure, at 800 K the critical radii for both LS and LS_2_ crystals are around 7 Å in excellent agreement with previous experimental estimations^45^.

The computed Helmholtz free energy barrier for nucleation (W*) of the LS_2_ crystal at 800 K is about 64 *kT*, agrees fairly well with the experimentally estimated ones, which are ranging from 35 and 50 *kT* at the temperature from 700 to 800 K^41^. The large variability associated with the free energy is due to the variability of the energy of frozen liquid structures generated through the standard MD quenching protocols. In fact, the fast quenching of the melts prevents the good sampling of the free energy surface and thus glass structures with different energies are easily generated^46^. Nevertheless, considering the unfavorable conditions in stoichiometry and structural similarities for the LS crystal in the bulk glass as discussed above, it is expected that the LS crystal can crystallize preferentially on the glass surface through the heterogeneous nucleation mechanism. Indeed, the energy barrier of heterogeneous nucleation $(W_{het}^{*} )$ is lower than that of the homogeneous one $(W_{hom}^{*} )$ according to a geometric factor $S\left( \theta \right)$, which depends on the wetting angle between the solid and liquid at the interface^47^. Further developments are required to extend the FESM method to investigate heterogeneous nucleation.

McKenzie et al.^40^ used a hybrid MD/MC approach to compute the free energy of nucleation of the LS and LS_2_ crystals inside the LS_2_ glass considered as an implicit medium. In their method, the nucleation free energy (NFE) was evaluated by considering three contributions: the cluster formation, the cluster to crystal transition and the cluster solvation energy. The thermodynamic barrier was estimated to be 40 kT and 34.5 kT for the LS_2_ and LS crystals, respectively.

They showed that the NFE is higher for the LS_2_ than for the LS at smaller cluster dimension, but it crossovers at around 4–5 formula units, and thus it was stated that the LS crystal first nucleates, and then it transforms to the LS_2_ with a thermodynamic barrier of 28.3 kT. A smaller free energy of nucleation at small cluster radius for the LS phase is also observed in our simulations, but since none of the two methods investigates the reactive pathway and the kinetics associated to an eventual crystalline phase transition from the LS to LS_2_, explicitly, it is difficult to make speculation on the phase transformation. Both our results and those by McKenzie et al.^40^ are in good agreement with the facts experimentally measured by Differential Thermal Analysis (DTA) and SEM observations by Soares et al.^29^. In fact, the DTA data has shown that two exothermic peaks appear at 615–680 °C and 750–800 °C, and these peaks are associated with the crystallization of the LS and LS_2_ phases, respectively, by XRD measurement. Morphological observations by SEM have also confirmed the occurrence of these two crystallization mechanisms since needle-like and granular crystals were detected in the microstructure of samples including LS and LS_2_, respectively. The authors have also found that, in the early stage of crystallization in the LS_2_ glass, both the LS and LS_2_ crystals nucleate simultaneously and independently^28^. Further, the analysis of the crystallization kinetics through the Johnson–Mehl–Avrami equation suggested that the surface crystallization (Avrami exponent *n* = 1) associated to the first peak rather than volume crystallization (Avrami exponent *n* = 3) associated to the second peak is dominant in the crystallization process. This is because the activation energy (225–275 kJ/mol) of the former is substantially lower than that of the latter (425–500 kJ/mol). Therefore, the LS crystal nucleates at the glass surface because favorable stoichiometric and thermodynamic conditions for its precipitation are present, whereas the LS_2_ crystal appears in the bulk of the glass.

The effect of the temperature on the nucleation free energy profile was investigated only for the nucleation of the LS_2_ crystal, as shown in Fig. 9b. Although error bars are relatively large, it is clear that both the critical size and the activation energy barrier increases with temperature as expected.

In conclusion of this section, it is worth summarizing advantages of the FESM method, despite the not negligible error associated with the calculation of the free energy of the glass state. Firstly, it should be emphasized that our method inherently includes the surface energy penalty during the creation of the melt/crystal interface and the strain energy at the interface. To the best of our knowledge, the surface energy contribution was implicitly subsumed in the solvation term (parameterized for a particular system) of the hybrid MC method used by McKenzie et al.^40,48^ The MC simulation highlighted that the strain energy term does not play an important role in nucleation above the glass transition temperature, thereby the contribution is usually neglected^49^.

The interfacial free energy associated to the formation of the melt/crystal interface for the LS_2_ nucleus with critical size using Eq. (5) calculated at 800 K and 1000 K is respectively 0.3 ± 0.1 and 0.4 ± 0.1 J/m^2^. These values are comparable to the value estimated by Fokin et al.^50^ when accounting both the temperature and size dependence of the crystal/liquid surface energy.

Moreover, as shown above, our approach can be used to compute the thermodynamic barrier for homogeneous crystal nucleation at different temperatures. This would be useful because the thermodynamic barrier has been shown to exhibit an unusual increase with a decrease in temperature below the maximum nucleation rate for a variety of oxide glass-forming liquids^49^. Although the internal elastic stresses arising from the density misfits between the crystal and liquid phases might play a role, the phenomenon is still not completely understood and should be object of future investigations^49^.

**Figure 10:**
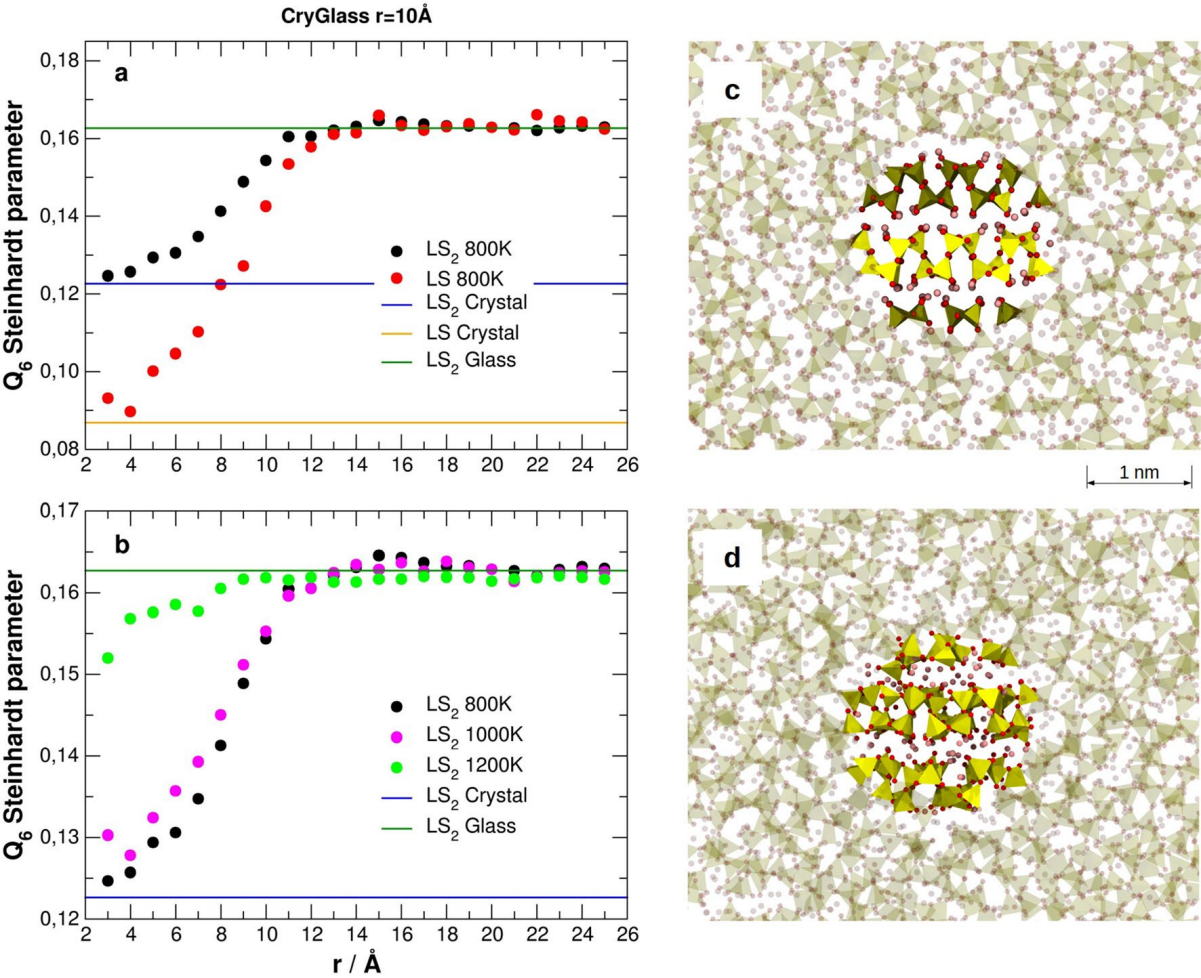
(**a**) Q_6_ Steinhardt parameter of silicon atoms from the center of the LS or LS_2_ crystal nuclei to the glass matrix at 800 K. (**b**) Computed local order Q_6_ parameter for silicon atoms from the center of the LS_2_ crystal nuclei (of 10 Å) to the glass matrix at 800 K, 1000 K and 1200 K. (**c**) Glass with LS_2_ composition with a nucleus of Li_2_Si_2_O_5_ crystal with radius of 10 Å equilibrated at 800 K. (**d**) Glass with LS_2_ composition with a nucleus of Li_2_Si_2_O_5_ crystal with radius of 10 Å equilibrated at 1200 K.

The approach is applicable to any system of interest since the parameters needed are only the interatomic force field for MD simulations. An advantage with respect to the original seeding method^30^ is that it allows extracting thermodynamic parameters of the nucleation process in high viscous liquids. Finally, all-atomistic MD simulations can provide information on the structure of the melt/crystal interface, which are hardly accessible from experiments. For instance, Fig. 10a shows the computed local order Q_6_ Steinhardt parameter (see ESI for definition) of silicon atoms from the center of the LS and LS_2_ crystal nuclei (of radius 10 Å shown in Fig. 10c) to the glass matrix at 800 K. The gradual change of Q_6_ Steinhardt parameter demonstrates gradual structural transition from the crystal to the glass (melt) structure and the profile allows us to measure the interface thickness in both cases. The LS_2_ crystal/LS_2_ glass interface thickness is of about 4 Å and less than that of the LS crystal/LS_2_ glass, which is about 8 Å. This may be due to the greater flexibility of the Q^2^ chains in the LS crystal that easily lose the perfect order at the interface with the glass. In Fig. 10b, the Q_6_ parameter was compared at 800 K, 1000 K and 1200 K to understand the effect of the temperature on the LS_2_ crystal/glass interface. The figure shows that the interface slightly enlarges in about 1–2 Å at 1000 K, while the Q_6_ value in the cluster approaches to that in the glass matrix and the crystalline order is almost lost at 1200 K. However, a visual inspection according to Fig. 10d reveals that the layered structure of the crystal is still maintained as well as the original topology of the silicate network and no Si–O bonds break. Only disordered crystalline structure is observed.

## Conclusions

Classical MD simulations were used to shed light on the crystal nucleation process inside and at the surface of the stoichiometric lithium disilicate glasses. In this work, we employed a modified cluster-exploring algorithm to detect subnano-scale nuclei in the initial crystallization stage, and the free energy calculation using a modified seeding method for the first time in multicomponent oxide glasses. Our simulations suggest that lithium disilicate and lithium metasilicate crystals nucleate independently. The former would appear homogeneously from the bulk, whereas the latter can heterogeneously nucleate only on the glass surface where favorable exogenous conditions are present. Therefore, the failure of CNT in predicting the magnitude of the steady state nucleation rate is not due to the formation of metastable phases as suggested in some of earlier investigations^27–29^.

As observed in previous works, homogeneous nucleation is associated with the similarity in intermediate range-order morphology of modifier cations between the crystal and the glass. In fact, the Li–Li second dipolar moments computed on the bulk glass model is very close to that of the LS_2_ crystal. The structural analysis using the improved cluster algorithm reveals that embryos of about 4–4.5 Å of radius with stoichiometry and structure very similar to those of the LS_2_ and LS crystals nucleate during glass formation both in the bulk and on the surface. The presence of such embryos in the MD-derived structural models is considered an indication of which crystal phase can crystallize from the glass forming liquid. The method thus hopefully serves the possibility to predict crystallization in the other multicomponent glasses in future investigations. Another interesting finding is that the addition of the nucleating agent P_2_O_5_ leads isolated orthophosphates units formation that attracts Li ions and promotes Li_3_PO_4_ embryos in the glass. This phenomenon facilitates crystallization of lithium metasilicate crystals on the surface where chains of Q^2^ silica species surrounded by Li ions are dominant rather than inside the bulk.

The FESM method allowed us to compute the free energy of nucleation for the LS_2_ and LS crystals in the stoichiometric LS_2_ glass and to investigate the effect of the temperature. The critical radii obtained are in excellent agreement with experimental estimations, and the activation energy barriers are comparable to the experimental data. This seems to confirm that both crystal phases form obeying the CNT if the capillarity approximation is overcome. That is, those thermodynamic properties such as the interfacial free energy assume size dependent values as inherently assumed in the FESM approach. Therefore, we confirm that the FESM is a powerful approach to study nucleation in glass, for instance to predict which crystals would nucleate more easily or extract key thermodynamic parameters as a function of nucleus size, temperature and pressure.

## Supplementary information

Supplementary file1

## Data Availability

The data that supports the findings of this work are available from the corresponding author upon reasonable request.

## References

[1] Hall KW (2018). Does local structure bias how a crystal nucleus evolves?. J. Phys. Chem. Lett..

[2] Sosso GC (2016). Crystal nucleation in liquids: open questions and future challenges in molecular dynamics simulations. Chem. Rev..

[3] Sosso GC (2018). Unravelling the origins of ice nucleation on organic crystals. Chem. Sci..

[4] Brehault A (2018). Compositional dependence of solubility/retention of molybdenum oxides in aluminoborosilicate-based model nuclear waste glasses. J. Phys. Chem. B.

[5] Jantzen CM, Brown KG, Pickett JB (2010). Durable glass for thousands of years. Int. J. Appl. Glass Sci..

[6] Höland W, Beall GH, Somiya S (2013). Chapter 5.1—Glass–ceramics. Handbook of Advanced Ceramics.

[7] Fokin VM, Zanotto ED, Yuritsyn NS, Schmelzer JWP (2006). Homogeneous crystal nucleation in silicate glasses: a 40 years perspective. J. Non-Cryst. Solids.

[8] Cormier L (2014). Nucleation in glasses—new experimental findings and recent theories. Procedia Mater. Sci..

[9] Zanotto ED (2013). Glass crystallization research—a 36-year retrospective. Part I, fundamental Studies. Int. J. Appl. Glass Sci..

[10] Varshneya AK (2006). Fundamentals of Inorganic Glasses.

[11] Karpukhina N, Hill RG, Law RV (2014). Crystallisation in oxide glasses—a tutorial review. Chem. Soc. Rev..

[12] Cormier L (2011). Investigation of the role of nucleating agents in MgO–SiO_2_–Al_2_O_3_–SiO_2_–TiO_2_ glasses and glass-ceramics: a XANES study at the Ti K- and L2,3-edges. Cryst. Growth Des..

[13] Rampf M, Dittmer M, Ritzberger C, Höland W (2016). Controlled parallel crystallization of lithium disilicate and diopside using a combination of internal and surface nucleation. Front. Mater..

[14] Khalkhali Z, Yekta BE, Marghussian VK (2012). Mechanical and chemical properties of Zr and P-doped lithium disilicate glass ceramics in dental restorations. Int. J. Appl. Ceram. Technol..

[15] Fokin VM (2010). Sodium fluoride solubility and crystallization in photo-thermo-refractive glass. J. Am. Ceram. Soc..

[16] Longstaffe JG (2008). Intermediate-range order of alkali disilicate glasses and its relation to the devitrification mechanism. J. Phys. Chem. C.

[17] Zanotto ED, Tsuchida JE, Schneider JF, Eckert H (2015). Thirty-year quest for structure–nucleation relationships in oxide glasses. Int. Mater. Rev..

[18] Lusvardi G (2005). A computational tool for the prediction of crystalline phases obtained from controlled crystallization of glasses. J. Phys. Chem. B.

[19] Zanotto ED, Fokin VM (2003). Recent studies of internal and surface nucleation in silicate glasses. Philos. Trans. R. Soc. Lond. Ser. Math. Phys. Eng. Sci..

[20] Niu H, Piaggi PM, Invernizzi M, Parrinello M (2018). Molecular dynamics simulations of liquid silica crystallization. Proc. Natl. Acad. Sci..

[21] Prado SCC, Rino JP, Zanotto ED (2019). Successful test of the classical nucleation theory by molecular dynamic simulations of BaS. Comput. Mater. Sci..

[22] Zanotto ED, Leite MLG (1996). The nucleation mechanism of lithium disilicate glass revisited. J. Non-Cryst. Solids.

[23] Iqbal Y, Lee WE, Holland D, James PF (1999). Crystal nucleation in P2O5-doped lithium disilicate glasses. J. Mater. Sci..

[24] Deubener J, Brückner R, Sternitzke M (1993). Induction time analysis of nucleation and crystal growth in di- and metasilicate glasses. J. Non-Cryst. Solids.

[25] Burgner LL, Lucas P, Weinberg MC, Soares PC, Zanotto ED (2000). On the persistence of metastable crystal phases in lithium disilicate glass. J. Non-Cryst. Solids.

[26] Burgner LL, Weinberg MC, Lucas P, Soares PC, Zanotto ED (1999). XRD investigation of metastable phase formation in Li_2_O–2SiO_2_ glass. J. Non-Cryst. Solids.

[27] Bischoff C, Eckert H, Apel E, Rheinberger VM, Höland W (2011). Phase evolution in lithium disilicate glass–ceramics based on non-stoichiometric compositions of a multi-component system: structural studies by 29Si single and double resonance solid state NMR. Phys. Chem. Chem. Phys..

[28] Soares PC, Zanotto ED, Fokin VM, Jain H (2003). TEM and XRD study of early crystallization of lithium disilicate glasses. J. Non-Cryst. Solids.

[29] Soares RS, Monteiro RCC, Lima MMRA, Silva RJC (2015). Crystallization of lithium disilicate-based multicomponent glasses—effect of silica/lithia ratio. Ceram. Int..

[30] Espinosa JR, Vega C, Valeriani C, Sanz E (2016). Seeding approach to crystal nucleation. J. Chem. Phys..

[31] Tanaka H (2010). Bond orientational ordering in a metastable supercooled liquid: a shadow of crystallization and liquid–liquid transition. J. Stat. Mech. Theory Exp..

[32] Pedone A, Malavasi G, Menziani MC, Cormack AN, Segre U (2006). A new self-consistent empirical interatomic potential model for oxides, silicates, and silica-based glasses. J. Phys. Chem. B.

[33] Zheng X, Wen G, Song L, Huang X (2008). Effects of P2O5 and heat treatment on crystallization and microstructure in lithium disilicate glass ceramics. Acta Mater..

[34] Pedone A (2009). Properties calculations of silica-based glasses by atomistic simulations techniques: a review. J. Phys. Chem. C.

[35] Smith W, Forester TR (1996). DL_POLY_2.0: a general-purpose parallel molecular dynamics simulation package. J. Mol. Graph..

[36] Allen MP, Allen MP, Tildesley DJ, Tildesley DJ (1989). Computer Simulation of Liquids.

[37] Lodesani F (2020). Structural origins of the mixed alkali effect in alkali aluminosilicate glasses: molecular dynamics study and its assessment. Sci. Rep..

[38] Yip S, Li J, Tang M, Wang J (2001). Mechanistic aspects and atomic-level consequences of elastic instabilities in homogeneous crystals. Mater. Sci. Eng. A.

[39] Sapozhnikov FA, Ionov GV, Dremov VV (2008). An adaptive template method for analyzing crystal structures and defects in molecular dynamics simulations of high-rate deformations. Russ. J. Phys. Chem. B.

[40] McKenzie ME, Mauro JC (2018). Hybrid Monte Carlo technique for modeling of crystal nucleation and application to lithium disilicate glass–ceramics. Comput. Mater. Sci..

[41] Fokin VM (2016). Crystal nucleation in glass-forming liquids: variation of the size of the ‘structural units’ with temperature. J. Non-Cryst. Solids.

[42] Deubener J (2005). Structural aspects of volume nucleation in silicate glasses. J. Non-Cryst. Solids.

[43] Mastelaro VR, Zanotto ED, Lequeux N, Cortès R (2000). Relationship between short-range order and ease of nucleation in Na_2_Ca_2_Si_3_O_9_, CaSiO_3_ and PbSiO_3_ glasses. J. Non-Cryst. Solids.

[44] Van Vleck JH (1948). The dipolar broadening of magnetic resonance lines in crystals. Phys. Rev..

[45] Sycheva GA (2018). Homogeneous and heterogeneous crystal nucleation in glass of the Li_2_O–SiO_2_ system. Glass Phys. Chem..

[46] Urata S (2019). An efficient computational procedure to obtain a more stable glass structure. J. Chem. Phys..

[47] Kelton KF, Ehrenreich H, Turnbull D (1991). Crystal Nucleation in Liquids and Glasses. Solid State Physics.

[48] McKenzie ME (2018). Implicit glass model for simulation of crystal nucleation for glass–ceramics. NPJ Comput. Mater..

[49] Abyzov AS, Fokin VM, Rodrigues AM, Zanotto ED, Schmelzer JWP (2016). The effect of elastic stresses on the thermodynamic barrier for crystal nucleation. J. Non-Cryst. Solids.

[50] Fokin VM, Zanotto ED (2000). Crystal nucleation in silicate glasses: the temperature and size dependence of crystal/liquid surface energy. J. Non-Cryst. Solids.

