# Supplementary material for: Disclosing crystal nucleation mechanism in lithium disilicate glass through molecular dynamics simulations and free-energy calculations
Source: Sci Rep. 2020 Oct 20;10:17867. doi: 10.1038/s41598-020-74764-9 (PMC7576157; doi:10.1038/s41598-020-74764-9)
Supplement: Supplementary file 1 — Supplementary file1 [file 41598_2020_74764_MOESM1_ESM.pdf]

Supporting Information

Disclosing Crystal Nucleation Mechanism in Lithium Disilicate Glass through Molecular Dynamics Simulations and Free-Energy Calculations.

Federica Lodesani [a], Maria Cristina Menziani [a], Kei Maeda [b], Yoichi Takato [c], Shingo Urata [c] and Alfonso Pedone [a,*]

[a)] *Department of Chemical and Geological Sciences, University of Modena and Reggio Emilia, via G. Campi 103, 41125, Modena, Italia*

[b)] *Materials Integration Laboratories, AGC Inc., Yokohama, Kanagawa 221-8755, Japan*

[c)] *Innovative Technology Laboratories, AGC Inc., Yokohama, Kanagawa 221-8755, Japan*

Computational Details.

Table S1. Number of atomic species in the simulation boxes, cubic box side (L_{box}) and experimental density used to determine it.

	Li	Si	P	O	L_{box} (Å)	Density (g/cm^3)
**LS_2 Glass**	3000	3000	-	7500	54.26	2.34
**LS_2P1 Glass**	2640	2640	80	6800	52.41	2.35

Force-Field.

In this work we have used a modification of the PMMCS force field which is based on a rigid ionic model, with partial charges to handle the partial covalency of silicate systems and is given by the combination of a long-range Coulomb potential with a short-range Morse function for the Li-O, Si-O, P-O and O-O interactions, a repulsive part of the Buckingham potential for the Li-Li and Si-Li interactions and a repulsive contribution of the form B/r^{12}, necessary to prevent atomic collapse at high temperature and pressure used for the interactions modelled with the Morse function:

$$U(r_{ij}) = \frac{z_i z_j e^2}{r_{ij}} + D_{ij}\left[\left(1 - e^{-a_{ij}\left(r_{ij}-r_{ij}^0\right)}\right)^2 - 1\right] + \frac{B_{ij}}{r_{ij}^{12}} + A_{ij} e^{-\frac{r_{ij}}{\rho_{ij}}} \qquad (1)$$

The charges $z_{i,j}$ are fixed and consistent with the value of -1.2e for oxygen (thus z_{Si}=2.4, z_{Li}=0.6 etc…). D_{ij}, a_{ij} and r_{ij}^0 are fitting parameters connected respectively to the dissociation energy, curvature ($2Da^2$) and reference minimum position of the Morse function.

The original version of the PMMCS force-field was constituted by only pair-wise interactions as in the first three terms of eq. 1. However, since it is known that the inclusion of only pair-wise interactions leads to structure with large overestimation of the intertetrahedral angles T-O-T we have included a three body term to better reproduce the Si-O-Si, Si-O-P and P-O-P intertetrahedral angles. The screened harmonic exponential decaying three-body function as been used:

$$U(\theta_{ijk}) = \frac{k_{ijk}}{2}\left(\theta_{ijk} - \theta_{ijk,0}\right)^2 exp\left[-\left(\frac{r_{ij}}{\rho_1} + \frac{r_{ik}}{\rho_2}\right)\right] \qquad (2)$$

Where k_{ijk}, $\theta_{ijk,0}$, ρ_1 and ρ_2 are parameters connected to the force constant, reference angle of the *i*-*j*-*k* triplet and the decay function along the i-j and i-k bonds.

The parameters have been fitted using as training set the PES of the $(HO)_3Si$-O-Si-$(OH)_3$, $(HO)_3Si$-O-PO-$(OH)_2$ and $(HO)_2$-OP-O-PO-$(OH)_2$ dimers computed as a function of the intertetrahedral angles at the PBE0/6-311+G(d,p) level using the Gaussian09 code.[4]

The A and ρ parameters for the Li-Li and Li-Si interactions have been fitted using the structure of the $Li_2Si_2O_5$ and Li_2SiO_3 crystal. All the parameters are reported in **Table S2** and **S3**.

Table S2. Interatomic potential parameters for equation (1).*

**Pairs**	**D_{ij} (eV)**	**a_{ij} (Å^{-2})**	**r_{ij}^0 (Å)**	**B_{ij} (eV Å^{12})**
**O-O**	0.042395	1.379316	3.618701	22.0
**Si-O**	0.340554	2.006700	2.100000	1.0
**P-O**	0.831326	2.58583	1.77079	1.0
**Li-O**	0.001114	3.4295	2.68136	2.0
	**A_{ij} (eV)**	**ρ (Å)**		
**Li-Li**	176990.1	0.1736		

Table S3. Three-body interatomic potential parameters.

	**k_{jik} (ev / rad^2)**	**θ^0_{jik}**	**ρ_1 (Å)**	**ρ_2 (Å)**
**Si – O – Si**	16.9	109.47°	1.00	1.00
**Si – O – P**	32.4	75.0°	1.00	1.00
**P – O – P**	34.4	81.0°	1.00	1.00

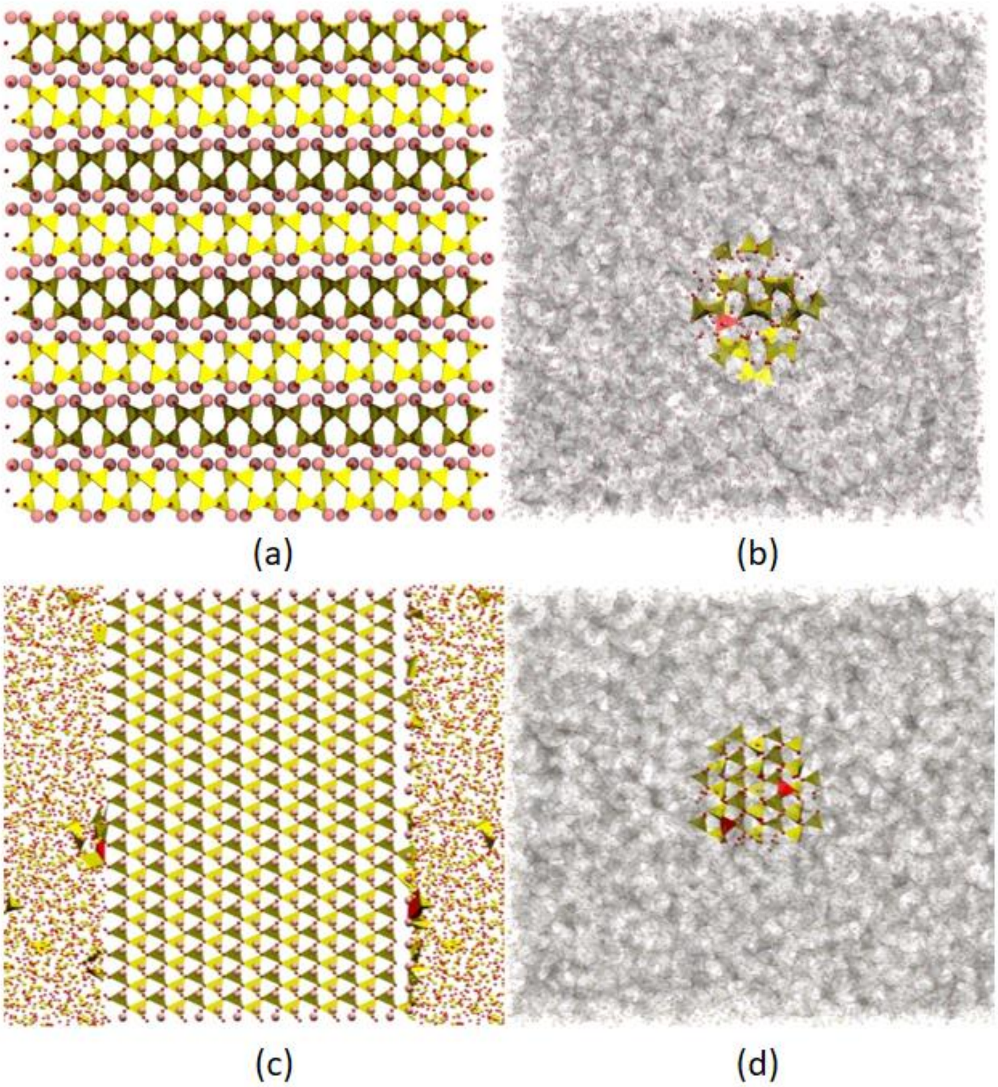

Figure S1. (a) The starting box ($Li_2Si_2O_5$ crystal) and b) the final box with a LS_2 crystal nucleus (8Å) embedded into the LS_2 glass matrix. (c) The starting and b) the final box with a LS crystal nucleus (8Å) embedded into the LS_2 glass matrix. Yellow tetrahedral represent silicon whereas red and pink spheres represent oxygen and lithium ions.

Table S4. Q^n distributions and network connectivity (NC) of the silica network for the LS_2 and LS_2P1 glasses.

	**Q^1**	**Q^2**	**Q^3**	**Q^4**	**NC**
**LS_2 Glass**	2.2	21.0	51.3	25.4	3.0
**LS_2P1 Glass**	1.4	18.7	49.4	30.5	3.1

Steinhardt Local Order Parameters

The Steinhardt order parameters has been widely used to distinguish between the liquid or glassy state to one or more crystal phases.[9]

The *l*-order Steinhardt parameter for atom *i* is defined by:

$$Q_l(i) = \sqrt{\frac{4\pi}{2l+1}\sum_{m=-l}^{l}|q_{lm}(i)|^2}$$

where

$$q_{lm}(i) = \frac{1}{N(i)}\sum_{j=1}^{N(i)} Y_{lm}(r_{ij})$$

Where the index *j* runs on the N(*i*) neighbors of atom *i* within a certain cutoff. $Y_{lm}(r_{ij})$ is a spherical harmonic with a vector displacement $\boldsymbol{r}_{ij}$ from atoms *i* and *j*. The subscript *l* is a free integer parameter that confines *m* between –*l* and +*l*.

References

(1) Pedone, A. Properties Calculations of Silica-Based Glasses by Atomistic Simulations Techniques: A Review. *J. Phys. Chem. C* **2009**, *113* (49), 20773–20784. https://doi.org/10.1021/jp9071263.

(2) Smith, W.; Forester, T. R. DL_POLY_2.0: A General-Purpose Parallel Molecular Dynamics Simulation Package. *J. Mol. Graph.* **1996**, *14* (3), 136–141.

(3) Allen, M. P.; Allen, M. P.; Tildesley, D. J.; Tildesley, D. J. *Computer Simulation of Liquids*; Clarendon Press, 1989.

(4) *M. J. Frisch, G. W. Trucks, H. B. Schlegel, G. E. Scuseria, M. A. Robb, J. R. Cheeseman, G. Scalmani, V. Barone, B. Mennucci, G. A. Petersson, H. Nakatsuji, M. Caricato, X. Li, H. P. Hratchian, A. F. Izmaylov, J. Bloino, G. Zheng, J. L. Sonnenberg, M. Hada, M. Ehara, K. Toyota, R. Fukuda, J. Hasegawa, M. Ishida, T. Nakajima, Y. Honda, O. Kitao, H. Nakai, T. Vreven, J. A. Montgomery, Jr., J. E. Peralta, F. Ogliaro, M. Bearpark, J. J. Heyd, E. Brothers, K. N. Kudin, V. N. Staroverov, R. Kobayashi, J. Normand, K. Raghavachari, A. Rendell, J. C. Burant, S. S. Iyengar, J. Tomasi, M. Cossi, N. Rega, J. M. Millam, M. Klene, J. E. Knox, J. B. Cross, V. Bakken, C. Adamo, J. Jaramillo, R. Gomperts, R. E. Stratmann, O. Yazyev, A. J. Austin, R. Cammi, C. Pomelli, J. W. Ochterski, R. L. Martin, K. Morokuma, V. G. Zakrzewski, G. A. Voth, P. Salvador, J. J. Dannenberg, S. Dapprich, A. D. Daniels, Ö. Farkas, J. B. Foresman, J. V. Ortiz, J. Cioslowski, and D. J. Fox, Gaussian 09 (Gaussian, Inc., Wallingford CT, 2009).*

(5) Longstaffe, J. G.; Werner-Zwanziger, U.; Schneider, J. F.; Nascimento, M. L. F.; Zanotto, E. D.; Zwanziger, J. W. Intermediate-Range Order of Alkali Disilicate Glasses and Its Relation to the Devitrification Mechanism. *J. Phys. Chem. C* **2008**, *112* (15), 6151–6159. https://doi.org/10.1021/jp711438v.

(6) Puls, S. P.; Eckert, H. Spatial Distribution of Lithium Ions in Glasses Studied by 7Li{6Li} Spin Echo Double Resonance. *Phys. Chem. Chem. Phys.* **2007**, *9* (30), 3992–3998. https://doi.org/10.1039/B705338J.

(7) Van Vleck, J. H. The Dipolar Broadening of Magnetic Resonance Lines in Crystals. *Phys. Rev.* **1948**, *74* (9), 1168–1183. https://doi.org/10.1103/PhysRev.74.1168.

(8) Lusvardi, G.; Malavasi, G.; Menabue, L.; Menziani, M. C.; Pedone, A.; Segre, U. A Computational Tool for the Prediction of Crystalline Phases Obtained from Controlled Crystallization of Glasses. *J. Phys. Chem. B* **2005**, *109* (46), 21586–21592. https://doi.org/10.1021/jp0546857.

(9) Quigley, D.; Rodger, P. M. A Metadynamics-Based Approach to Sampling Crystallisation Events. *Mol. Simul.* **2009**, *35* (7), 613–623. https://doi.org/10.1080/08927020802647280.
